# Rash and cholestatic liver injury caused by methimazole in a woman with Turner syndrome and Graves’s disease: a case report and literature review

**DOI:** 10.1186/s12902-021-00819-1

**Published:** 2021-09-03

**Authors:** Jinhui Zeng, Fangtao Luo, Zhihua Lin, Yinghong Chen, Xiaoyun Wang, Yuanhao Song

**Affiliations:** Department of Endocrinology, The Second Affiliated Hospital of Xiamen Medical College, 361021 Xiamen, Fujian China

**Keywords:** Turner syndrome, Graves' disease, Methimazole, Rash, Cholestatic liver injury

## Abstract

**Background:**

Rash and cholestatic liver injury caused by methimazole (MMI) in patients with Turner syndrome (TS) and Graves’s disease (GD) are rarely reported, and there is a paucity of reports on the management of this condition. It is not clear whether propylthiouracil (PTU) can be used as a safe alternative in this case.

**Case presentation:**

A 37-year-old woman was admitted to our hospital with rash, severe pruritus and a change in urine colour after 2 months of GD treatment with MMI. Physical examination showed rash scattered over the limbs and torso, mild jaundice of the sclera and skin, short stature, facial moles, immature external genitals and diffuse thyroid gland enlargement. Liver function tests indicated an increase in total bilirubin, direct bilirubin, total bile acid, glutamic pyruvic transaminase, glutamic oxaloacetic transaminase and alkaline phosphatase. The level of sex hormones suggested female hypergonadotropic hypogonadism. The karyotype of peripheral blood was 46, X, i(X)(q10)/45, X. After excluding biliary obstruction and other common causes of liver injury, combined with rash and abnormal liver function following oral administration of MMI, the patient was diagnosed as having TS with GD and rash and cholestatic liver injury caused by MMI. MMI was immediately discontinued, and eleven days after treatment with antihistamine and hepatoprotective agents was initiated, the rash subsided, and liver function returned to nearly normal. Because the patient did not consent to administration of ^131^I or thyroid surgery, hyperthyroidism was successfully controlled with PTU. No adverse drug reactions were observed after switching to PTU.

**Conclusions:**

While patients with TS and GD are undergoing treatment with MMI, their clinical manifestations, liver functions, and other routine blood test results should be closely monitored. When patients with TS and GD manifest adverse reactions to MMI such as rash and cholestatic liver injury, it is necessary to discontinue MMI and treat with antihistamine and hepatoprotective agents. After the rash subsides and liver function returns to nearly normal, PTU can effectively control hyperthyroidism without adverse drug reactions.

## Background

TS is a rare chromosomal abnormality with complete or partial deletion of the X chromosome [1], and 20–50 girls per 100,000 live births are affected [2]. Women with TS have a significantly increased risk of autoimmune diseases, the most common of which is autoimmune thyroid disease (ATD) [3, 4]. Moreover, among girls with TS, the prevalence of GD was significantly lower than that of Hashimoto’s thyroiditis (TH) [5]. There seems to be no difference in the clinical course of GD patients with or without TS [6, 7] and in the remission rate after the start of MMI treatment and the recurrence rate after MMI withdrawal at the end of the first treatment cycle between GD patients with or without TS [6].

Rash and cholestatic liver injury caused by MMI in patients with TS and GD are rarely reported, and there is a paucity of reports on the management of this condition. It is not clear whether PTU can be used as a safe alternative in this case.

### Case presentation

A 37-year-old woman was admitted to the hospital on September 14, 2020, due to rash, severe pruritus and a change in urine colour. Six months prior, the patient developed palpitation, fatigue and weight loss. Two months prior, thyroid function test results were as follows: thyroid stimulating hormone (TSH) 0.01 µIU/ml (normal range: 0.27– 4.20 µIU/ml), free triiodothyronine (FT3) 14.01 pmol/L (normal range: 3.1–6.8 pmol/L), free thyroxine (FT4) 49.77 pmol/L (normal range: 12.0–22.0 pmol/L), and anti-thyrotropin receptor antibody (TRAb) 37.79 IU/L (normal range: 0-1.75 IU/L). The patient were diagnosed as GD. Before treatment, hepatic function test results was as follows: total bilirubin (TBIL) 6.90µmol/L (normal range, 3.0–25.0µmol/L), direct bilirubin (DBIL) 2.90µmol/L (normal range, 0.0–6.0µmol/L), alkaline phosphatase (ALP) 81.00IU/L (normal range, 35–100IU/L), gamma-glutamyl transferase (GGT) 32.00IU/L (normal range, 7–45IU/L), aspartate aminotransferase (AST) 27.50IU/L (normal range, 13–35IU/L), and alanine aminotransferase (ALT) 21.70IU/L (normal range, 7–40IU/L). Other routine blood tests were normal. A local doctor prescribed 10 mg MMI and 10 mg propranolol three times a day. Two months later, the patient began to develop rash, severe pruritus, and noticed a change in urine colour before she was referred to our department for consultation. The patient presented with primary amenorrhoea and still had no menstruation at the age of 20. At that time, she started menstruating after 3 months of female hormone replacement therapy in a local hospital, but she stopped menstruating after discontinuing the drug on her own, and there was no further diagnosis or treatment. She was married at the age of 30 but was never pregnant. She had no family history of autoimmune diseases. Her body temperature was 36.6 °C, pulse rate was 71 beats/minute, respiratory rate was 20 breaths/min, blood pressure was 109/73 mmHg, and she was 140 cm tall, weighed 30 kg and her body mass index was 15.3 kg/m^2^. The rash was scattered over the limbs and torso. Mild jaundice was observed in the sclera and the skin. Numerous moles on the face and diffuse thyroid gland enlargement were also noted. Her external genitalia were underdeveloped for her age, and her breast and pubic hair was at Tanner stage 2. There were no webbed neck or bone deformities.

Laboratory tests and imaging studies were performed. Thyroid function test results were as follows: TSH 0.013 µIU/ml (normal range: 0.27– 4.20 µIU/ml), FT3 4.90 pmol/L (normal range: 3.1–6.8 pmol/L), FT4 14.56 pmol/L (normal range: 12.0–22.0 pmol/L), and TRAb 12.45 IU/L (normal range: 0-1.75 IU/L). Hepatic function test results were as follows: total bilirubin (TBIL) 43.80µmol/L (normal range, 3.0–25.0µmol/L), direct bilirubin (DBIL) 31.10µmol/L (normal range, 0.0–6.0µmol/L), alkaline phosphatase (ALP) 559.00IU/L (normal range, 35–100IU/L), gamma-glutamyl transferase (GGT) 412.00IU/L (normal range, 7–45IU/L), aspartate aminotransferase (AST) 71.90IU/L (normal range, 13–35IU/L), and alanine aminotransferase (ALT) 90.10IU/L (normal range, 7–40IU/L). Albumin, prothrombin time, partial thrombin time and blood cell count were all in the normal range. The serology of hepatitis viruses A, B, C, D and E were all negative. Autoantibody profiles of primary biliary cirrhosis and autoimmune hepatitis, including anti-mitochondrial M2 (AMA-M2) autoantibodies, anti-gp210 antibodies, anti-sp100 antibodies, anti-LC1 antibodies, anti-liver kidney microsomal antibody type 1 (LKM-1), and anti-soluble liver antigen (SLA) antibodies, were all negative. Abdominal ultrasound showed no significant changes in the liver, pancreas or spleen and no signs of biliary dilatation. After excluding biliary obstruction and other common causes of liver injury, combined with abnormal liver function following oral administration of MMI, the patient was diagnosed as drug-induced liver injury.

**Fig. 1:**
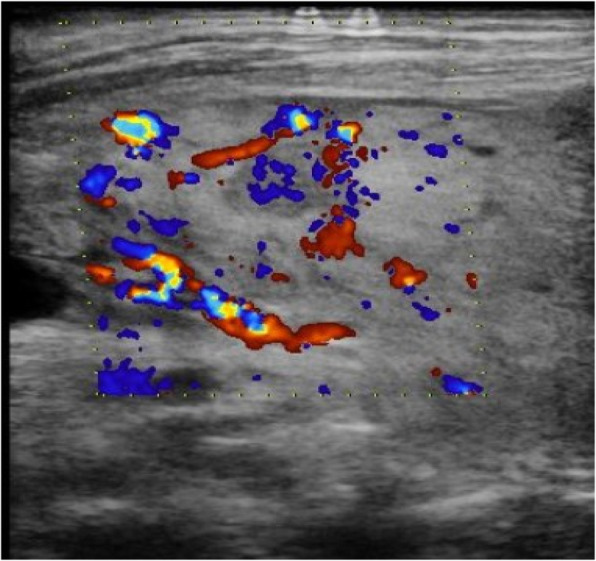
Color Doppler ultrasound of thyroid gland of the patient

**Fig. 2:**
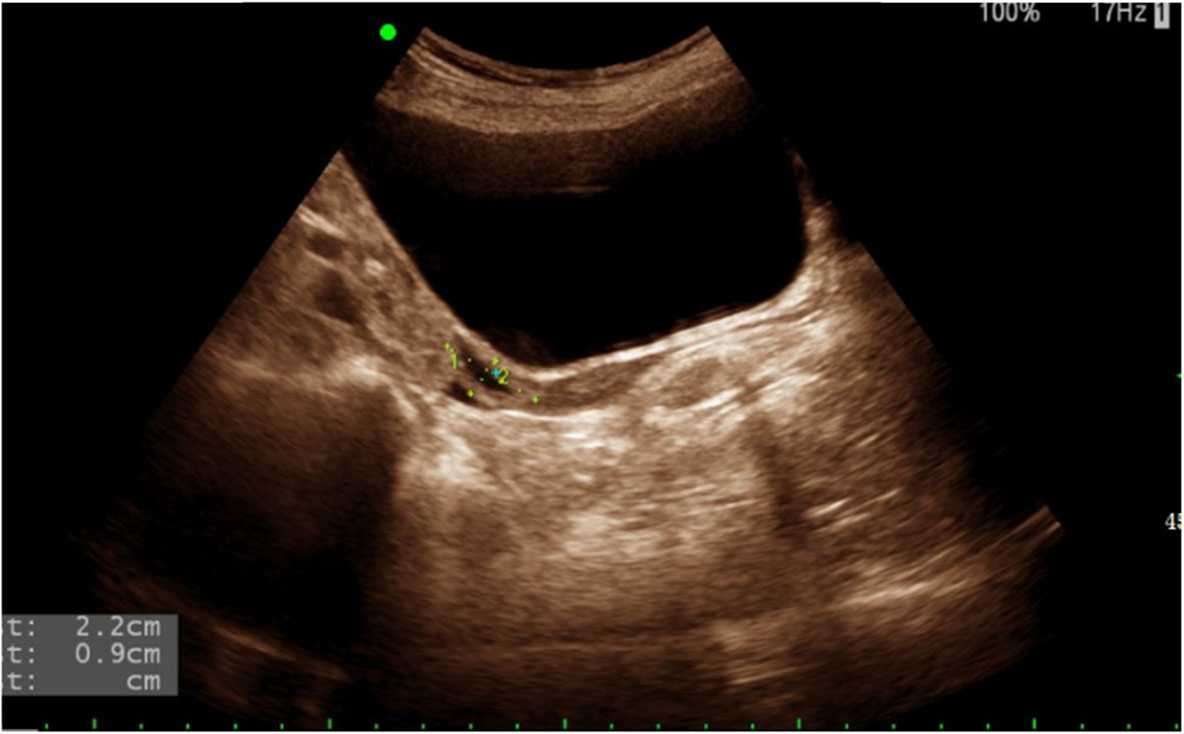
Pelvis ultrasonography of the patient. It showed immature uterus, not detecting bilateral ovaries

**Fig. 3:**
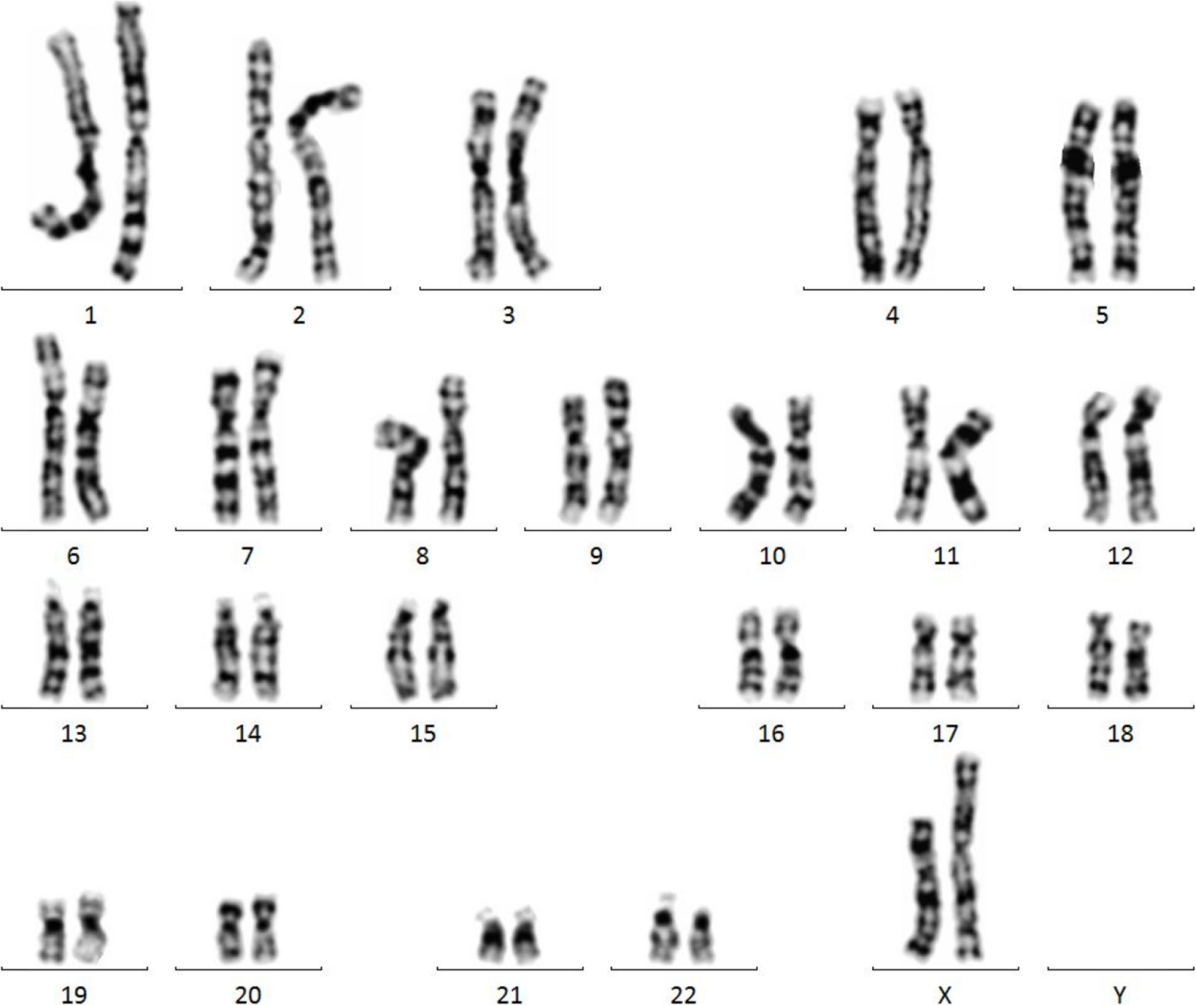
The karyotype of peripheral blood of the patient: 46, X, i(X)(q10)/45, X

Sex hormone test results were as follows: follicle-stimulating hormone (FSH) 89.88 mIU/ml, luteinizing hormone (LH) 29.11 mIU/ml, oestradiol (E2) < 73.00pmol/L, progesterone (P) 0.86 nmol/L, prolactin (PRL) 130.44 mIU/L, and testosterone (TSTO) 0.55 nmol/L. Diffuse enlargement of the thyroid gland was observed by colour Doppler ultrasound (Fig. 1). No intracardiac structural abnormalities or aortic stenosis were found by echocardiography. Electrocardiogram showed sinus rhythm, and the QT interval was 430 ms. Urinary tract ultrasound showed double renal pelvis. Pelvic ultrasound showed a small uterus, and ovary could not be detected on either side (Fig. 2). Bone mineral density (BMD) indicated severe osteoporosis (T-score: L1–4 BMD − 5.2, left femoral neck BMD − 4.3, right femoral neck BMD − 3.7). When biochemical markers of bone metabolism were measured, beta-CrossLaps(COX) was 1.81 ng/mL (normal range, 0.030–1.008 ng/mL), N-MID-Osteocalcin was 30.31 ng/mL (normal range, 11–43 ng/mL), total N-terminal propeptide of type I procollagen was 175.40 ng/mL (normal range, 15.13–76.31 ng/mL), total 25-OH-vitamin D was 10.94 ng/mL (normal range, > 20 ng/mL), and parathyroid hormone was 20.16 pg/mL (normal range, 14.9–56.9 pg/mL). Her karyotype was 46, X, i(X)(q10)/45, X (Fig. 3). Collectively, we diagnosed this patient as having TS with GD and rash and cholestatic liver injury caused by MMI.

Once a definite diagnosis was made, we immediately discontinued MMI. After 11 days of daily intravenous infusion of 1000 mg adenosylmethionine 1,4-butanedisulfonate and three times daily administration of 2 mg cyproheptadine hydrochloride tablets, the patient’s liver function gradually returned to nearly normal, rash and jaundice disappeared, and urine colour returned to normal (Table 1). At that time, we advised the patient to undergo either ^131^I injection or surgery for GD, but the patient refused either treatment. Consequently, hyperthyroidism was managed with 25 mg of PTU two times daily, and liver function was monitored continuously during the switch to PTU therapy. In the meantime, the patient received 0.6 g calcium carbonate, vitamin D3 tablets, and 0.25 µg calcitriol soft capsules daily to treat severe osteoporosis.

**Table 1:** Thyroid function and liver function during hospitalization

Parameters	Values(2020.09.14)	Values(2020.09.18)	Values (2020.09.25)	Reference ranges
FT3(pmol/L)	-	4.90	-	3.1–6.8
FT4(pmol/L)	-	14.56	-	12.0–22.0
TSH(uIU/ml)	-	0.013	-	0.27–4.20
TBIL(umol/L)	43.80	26.20	14.35	3.0–25.0
DBIL(umol/L)	31.10	21.80	7.60	0–6.0
ALP(IU/L)	559.00	371.00	227.00	35–100
GT(IU/L)	412.00	250.00	113.00	7–45
AST(IU/L)	71.90	32.70	41.00	13–35
ALT(IU/L)	90.10	33.40	22.00	7–40

After discharge, the patient’s general physical condition, routine blood tests and liver function were closely monitored at the outpatient clinic, and the dose of PTU was adjusted according to the patient’s thyroid function. The patient has been treated with 0.6 g calcium carbonate, vitamin D3 tablets, and 0.25 µg calcitriol soft capsules daily to treat severe osteoporosis after discharge. Three months after discharge, the patient received female hormone replacement therapy, which involved 2 mg of oestradiol valerate tablets daily for 11 days, 1 mg of oestradiol cyproterone tablets daily for 10 days, followed by 7 days of treatment interruption. The patient started menstruating after one course of female hormone replacement therapy. At the same time, PTU was reduced to 25 mg daily. Nearly eight months after discharge, the patient was still treated with 25 mg of PTU daily and was receiving female hormone replacement therapy, with normal thyroid and hepatic function (Table 2).

**Table 2:** Thyroid function and liver function after discharge

Parameters	Values(2020.10.17)	Values(2020.11.29)	Values(2021.01.29)	Values(2021.05.18)	Reference ranges
FT3(pmol/L)	-	4.35	4.71	5.13	3.1–6.8
FT4(pmol/L)	-	10.95	13.87	14.29	12.0–22.0
TSH(uIU/ml)	-	3.220	1.200	0.348	0.27–4.20
TBIL(umol/L)	7.67	13.78	5.50	17.60	3.0–25.0
DBIL(umol/L)	2.80	3.50	2.20	5.80	0–6.0
ALP(IU/L)	166.00	197.00	189.00	159.00	35–100
GT(IU/L)	38.00	65.00	49.00	36.00	7–45
AST(IU/L)	22.00	16.00	19.70	31.40	13–35
ALT(IU/L)	12.00	14.00	9.80	28.20	7–40

## Discussion and conclusions

TS is usually characterized by short stature, delayed puberty, ovarian hypoplasia, hypogonadism, infertility, congenital heart malformation, osteoporosis and autoimmune diseases such as ATD, type 1 diabetes and inflammatory bowel disease [1]. Women with TS are prone to autoimmune diseases, the most common being ATD, including HT and GD [3, 8, 9]. The incidence of HT is 11.1-25.2 % [8, 10, 11], and the incidence of GD is 0.4–1.8 % [7, 8, 10, 11]. A recent study has shown that patients with TS with karyotypes 45, X/46, isoXq and 45 X have a higher prevalence of complications, including ATD, than patients with mosaic 45, X/46, XX or Y chromosomes [12]. Thyroid function is more likely to deteriorate over time in young HT patients with TS [6], and there is a higher risk of developing GD [6, 13], therefore, thyroid status needs to be closely monitored during the clinical course of these patients. Unlike patients with TS and HT, patients with TS and GD do not show clinical and biochemical features distinct from GD patients without TS, but the incidence of GD is higher in TS patients [6]. Furthermore, a study found that there was no difference in the effective dose of MMI, the initial remission rate or the recurrence rate after withdrawal upon completing the first course of treatment in GD patients with TS compared with those in GD patients without TS [7].

Although long-term antithyroid drug treatment for GD is mostly safe, it can sometimes cause mild complications such as skin rash and even serious complications such as agranulocytosis, hepatotoxicity and vasculitis [14]. A recent meta-analysis showed that there was no difference in the incidence of elevated bilirubin, agranulocytosis, rash or urticaria between MMI and PTU in the treatment of hyperthyroidism [15]. However, PTU causes more severe hepatotoxicity and a higher incidence of hepatotoxicity than MMI. PTU is not recommended in children and it should be used with caution in adults [16]. The risk of severe hepatotoxicity is high, especially in the first three months of antithyroid drug therapy, and there is no difference in the type of severe hepatotoxicity caused by MMI and PTU [17]. ^131^I therapy is an effective alternative treatment if the patient does not tolerate antithyroid drugs due to severe hepatotoxicity [17]. When a mild skin reaction occurs during the treatment of GD with MMI, antihistamines can be administered without discontinuing MMI, but the antithyroid drugs should be discontinued immediately and actively treated with anti-allergic medications in severe cases; when mild cholestatic liver injury occurs, the dosage of MMI can be reduced while administering hepatoprotective drugs, but when the patient presents severe jaundice, MMI should be discontinued immediately to avoid liver failure [18].

A study has shown that it is safe to switch from one antithyroid drug to another when mild side effects are observed during treatment of GD. 34 % of patients who switch to PTU and 30 % of patients who switch to MMI presented side effects that are usually the same as the original type of side effects, while the rest of the patients had no complications [19]. It is not clear from this paper whether the clinical management of rash, pruritus and cholestatic liver damage in GD patients with TS is the same as that in GD patients without TS and whether PTU can be used as an alternative in this case. It was previously reported that, in patients with cholestatic hepatitis induced by MMI while undergoing treatment for GD, PTU was successfully used to control hyperthyroidism after MMI was discontinued while liver protection treatment was administered simultaneously [20]. However, the autoimmunity is enhanced in patients with TS, and there may be asymptomatic liver injury characterized by increases in ALT, AST and GGT alone [2]. Therefore, it is necessary to be cautious about using PTU when allergic reactions such as rash, pruritus, cholestatic liver injury and so on are observed. In addition, a study showed that TS patients with karyotype 45, X/46, isoXq and 46, X, r(X)/46, XX were more likely to develop liver dysfunction [12]. When such patients presents liver injury during treatment with antithyroid drugs, it may be more appropriate to switch to ^131^I and surgical therapy.

Following the EASL Clinical Practice Guidelines on drug-induced liver injury [21], rash and cholestatic liver injury caused by MMI in a patient with TS and GD were diagnosed. We immediately discontinued MMI and administered antihistamines and hepatoprotective treatment. The rash subsided and liver function gradually recovered. As the patient refused ^131^I or surgical therapy, she was treated with an alternative antithyroid mediation PTU, routine blood tests and liver function tests were regularly performed and the patient’s overall physical condition was closely monitored during treatment. Finally, hyperthyroidism was successfully controlled without adverse reactions such as rash, liver function damage, agranulocytosis, vasculitis and so on. It was previously reported that hyperthyroidism went into remission in a 17-year-old female with TS and GD after 18 months of treatment with MMI but recurred 9 months later, and thyroid function returned to normal after 2 years of retreatment with antithyroid drugs [22]. Similarly, another study showed that MMI was used successfully to control hyperthyroidism in a patient with TS and GD [23]. However, in another report, PTU failed to control hyperthyroidism in a patient with TS and GD and the patient had to undergo subtotal thyroidectomy [24]. In another study, PTU successfully controlled hyperthyroidism in a patient with TS and GD, but the patient ultimately had to be treated with ^131^I therapy due to vasculitis [25], and the researchers believe that TS patients are at risk of developing autoimmune diseases, which may increase the incidence of vasculitis and other autoimmune diseases during treatment with antithyroid drugs. PTU can cause not only clinically obvious, severe or even fatal liver injury but also induce skin-limited vasculitis or necrotizing skin injury [26]. TS can lead to liver injury characterized by asymptomatic elevation of ALT, AST, and GGT. Therefore, close monitoring of liver function and autoimmune diseases such as vasculitis should be performed when PTU is used to treat patients with TS and GD. Our patients had received oral PTUs for 8 months without any obvious adverse reactions. We expect that oral PTU can effectively control patients’ hyperthyroidism and fulfil the patient’s wishes not to receive ^131^I therapy or thyroid surgery.

A recent study has shown that GD individuals carrying the HLA-C*03:02 allele have an increased risk of MMI-induced liver injury. HLA-C*03:02 and HLA-A*02:01 are both susceptibility loci for MMI-induced cholestasis and mixed liver injury [27]. Another study has shown that carrying the HLA-DRB1*04:03 allele in the Han population is a high-risk factor for MMI-induced cutaneous adverse reactions such as urticaria, maculopapular eruption, and drug-induced hypersensitivity syndrome [28]. Due to the high price, the patient refused to undergo genetic analyses to determine whether she carried gene sequences related to susceptibility to adverse skin reactions to antithyroid drug and liver injury.

Our successful management of this patient owes to the following: (1) rapid confirmation of the diagnosis of TS with GD and cholestatic liver injury and rash caused by MMI; (2) timely discontinuation of MMI and liver protection and antihistamine therapy; (3) patient’s general condition, routine blood tests and liver function were closely monitored during treatment with PTU; (4) female hormone replacement therapy may have been beneficial as hepatoprotection. However, we also found room for improvement in our management: (1) patient failed to undergo more appropriate treatment such as ^131^I and thyroid surgery; (2) patient did not undergo genetic analysis to determine whether she carried gene sequences related to antithyroid drug-induced liver injury; (3) we failed to establish a multidisciplinary team to monitor the health of patients for comorbidities and complications; (4) we failed to document the skin rash with pictures.

Our case report is expected to fill the knowledge gap in the clinical management of rash and cholestatic liver injury caused by MMI in patients with TS and GD and demonstrates that hyperthyroidism can be effectively controlled without adverse reactions by switching to PTU in this situation. However, it is not clear whether our management experience can be extended to similar adolescent patients with TS and GD experiencing antithyroid drug-related adverse skin reactions and liver injury.

In conclusion, when patients with TS and GD are treated with MMI, their general conditions, liver function and routine blood tests should be closely monitored. When patients with TS and GD present rash and cholestatic liver injury due to MMI, it is necessary to discontinue MMI and administer antihistamine and liver protectives. After the rash subsides and liver function returns to nearly normal, PTU can effectively control hyperthyroidism without adverse drug reactions.

## Data Availability

The datasets used and analysed during the current study are available from the corresponding author on reasonable request.

## Abbreviations

MMI: Methimidazole
TS: Turner syndrome
GD: Graves’s disease
PTU: Propylthiouracil
ATD: Autoimmune thyroid disease
HT: Hashimoto’s thyroiditis
TSH: Thyroid stimulating hormone
FT3: Free triiodothyronine
FT4: Free thyroxine
TRAb: Anti-thyrotropin receptor antibody
TBIL: Total bilirubin
DBIL: Direct bilirubin
ALP: Alkaline phosphatase
GGT: Gamma-glutamyl transferase
AST: Aspartate aminotransferase
ALT: Alanine aminotransferase
BMD: Bone mineral density
